# Meal-Based Intervention on Health Promotion in Middle-Aged Women: A Pilot Study

**DOI:** 10.3390/nu15092108

**Published:** 2023-04-27

**Authors:** Jinyoung Shon, Yehee Seong, Yeji Choi, Yeri Kim, Mi Sook Cho, Eunhee Ha, Oran Kwon, Yuri Kim, Yoon Jung Park, Yangha Kim

**Affiliations:** 1 Department of Nutritional Science and Food Management, Ewha Womans University, Seoul 03760, Republic of Korea; shon.jinyoung.layla@gmail.com (J.S.);; 2 Graduate Program in System Health Science and Engineering, Ewha Womans University, Seoul 03760, Republic of Korea; 3 Graduate School of Clinical Biohealth, Ewha Womans University, Seoul 03760, Republic of Korea; sungyh0127@naver.com (Y.S.);; 4 Department of Environmental Medicine, Ewha Womans University College of Medicine, Seoul 07804, Republic of Korea

**Keywords:** middle-aged women, dietary intervention, health promotion

## Abstract

Middle-aged women belong to a risk group for metabolic dysregulation and menopausal symptoms, mainly due to a dramatic hormonal shift. Supplementation with functional compounds or a single nutrient has been dominantly explored as a nutritional approach for improving aging-related health parameters. However, a meal-based approach might be another strategy for promoting the overall health of the target population. This pilot study aimed to develop a meal-based intervention for middle-aged women and to evaluate its potential health benefits. Considering the nutrient intake status of Korean middle-aged women, diets enriched with four major nutrients (isoflavone, omega-3, fiber, and calcium) were designed and provided to forty-nine women aged 50 to 65 with mild levels of menopausal symptoms for 8 weeks. In the post-intervention phase, they showed reduced body weight and body fat, and improved biochemical metabolic parameters with decreased levels of cholesterol, low-density lipoprotein-cholesterol, ApoB, and fasting insulin. Moreover, bone resorption markers and menopause symptoms were lower in the post-intervention phase. In conclusion, the meal-based intervention might be a prominent strategy for overall health promotion in relatively healthy middle-aged women and further investigation is needed to test its efficacy with a randomized controlled study.

## 1. Introduction

Middle-aged women are considered a health risk group due to their undergoing a dramatic hormonal shift, so-called menopause, and subsequential metabolic shift [1]. Menopause is the cessation of the menstrual cycle and the loss of ovarian activity influences hormonal changes, including a decline in estrogen, progesterone, and estradiol (E2) and an increase in follicle-stimulating hormone (FSH) [2]. Several accompanying symptoms, including hot flashes, sweating, depression, fatigue, and sleep disorders, have been reported as causes of low quality of life (QoL) in middle-aged women [3]. Moreover, changes in ovarian hormones are associated with reduced bone density, unfavorable alterations in blood lipid parameters, and increased insulin resistance, resulting in an increased risk of developing obesity, metabolic syndrome, and cardiovascular disease (CVD) [4,5]. To prevent complications, it is necessary to improve the health status through various therapeutic strategies, including hormonal therapy, physical activity, lifestyle management, and a healthy diet rich in fiber and antioxidant nutrients [6].

Several reports have suggested the possibility of a nutritional intervention to promote health in middle-aged women. Supplementation with specific nutrients, including isoflavone, fiber, omega-3, and calcium, has been suggested to reduce the risk for chronic metabolic diseases and to alleviate menopausal symptoms in middle-aged women [7,8,9,10,11,12,13,14]. In a prospective study of 60 healthy postmenopausal women, hot flashes and night sweats were reduced after 60 mg of soy isoflavones was administered daily for 12 weeks [7], and isoflavone extracted from soybeans improved mood, vasomotor symptoms, and general menopausal symptoms in menopausal women [8]. In addition, isoflavones contributed to improved lipid profiles in postmenopausal women with mild hypercholesterolemia [9]. Dietary fiber intake is reported to reduce the risk of coronary heart disease in menopausal women through various mechanisms, such as improving blood lipid profiles and reducing blood pressure and insulin resistance [10,11]. Omega-3 supplementation for 6 months improved insulin resistance and reduced inflammatory indicators in postmenopausal women with a moderate risk of metabolic diseases [12]. Increased intake of eicosapentaenoic acid and docosahexaenoic acid for 12 months ameliorated bone resorption and improved lipid profiles with decreases in total cholesterol (TC) and low-density lipoprotein-cholesterol (LDL-C) concentration [13]. Furthermore, a cross-sectional study of menopausal women revealed that a high calcium intake was negatively correlated with CVD risk factors [14].

While the importance of investigations into the health benefits of individual nutrients is well-acknowledged in nutritional epidemiology, the current evidence regarding the supplementation of individual nutrients and health improvement in menopausal women is not conclusive [15,16,17,18,19,20,21]. In particular, the efficacy of dietary intervention on menopausal symptoms is controversial, unlike its effects on metabolic parameters. In a 12-week randomized controlled study, isoflavone-supplemented menopausal women showed alleviated frequency and degree of hot flashes [22]. By contrast, supplementation of dietary isoflavone or soy protein for 12 weeks did not show positive effects; rather, it caused negative side effects, such as abdominal distension [15,16]. In a 4-month intervention study, isoflavone supplementation of 100 mg/day in 80 menopausal women decreased the levels of TC and LDL and menopausal symptoms [23], while isoflavone-rich soy protein supplementation for 6 months did not affect menopausal symptoms [17]. In addition, several randomized controlled trials that examined the effect of omega-3 supplementation on menopausal symptoms reported no improvements in the frequency and severity of hot flashes, insomnia severity, sleep quality, and QoL [18,19,20,21]. These inconsistent results were caused by differences in the concentration, duration, and form of supplementation of individual nutrients, highlighting the importance of the overall intake of crucial nutrients.

Recently, meal-based interventions have emerged as an alternative strategy to reduce risks associated with metabolic dysregulation and menopausal symptoms [6]. Various approaches to meal planning (e.g., modification of the portion of macronutrients or glycemic index) and healthy dietary patterns (e.g., Mediterranean diet) can be effective in achieving healthy metabolic goals [24]. Additionally, the potential synergetic impacts of the complex combinations of individual nutrients or components in food and the possibility of long-term induction of positive interactions between nutrients by consuming meals are strong benefits of the meal-based intervention [25]. In this context, focusing on healthy eating patterns, rather than individual nutrient supplementation, is necessary to ensure menopausal women have adequate nutrient intakes. Despite the distinct benefits of meal-based interventions, few studies have evaluated the effect of meal-based interventions in menopausal women due to the methodological difficulty, including providing an individual with a full meal. Furthermore, most studies have evaluated the effects of dietary intervention in subjects with a disease, resulting in their unverified effects in healthy middle-aged women.

Therefore, this study aimed to develop a specific meal-based intervention targeting isoflavone, omega-3, fiber, and calcium for relatively healthy, low symptomatic middle-aged women and evaluate its effects on health improvement as a pilot study.

## 2. Materials and Methods

### 2.1. Study Design and Study Population

This study was designed as a pilot study with pre−post-intervention comparison, in which the 8-week intervention was preceded by a run-in period of 2 weeks. Informed consent was obtained from all subjects involved in the study after an explanation of its purpose and process of the study, and the subjects’ information was recorded in the Case Report Form. This study was reviewed and approved by the Ethics Committee of Ewha Womans University College of Medicine (EUMC 2020-02-047-005, approval date 18 December 2020) and conducted from July 2021 to September 2021 at Ewha Womans University Mok-dong Hospital, Seoul, Korea.

**Figure 1:**
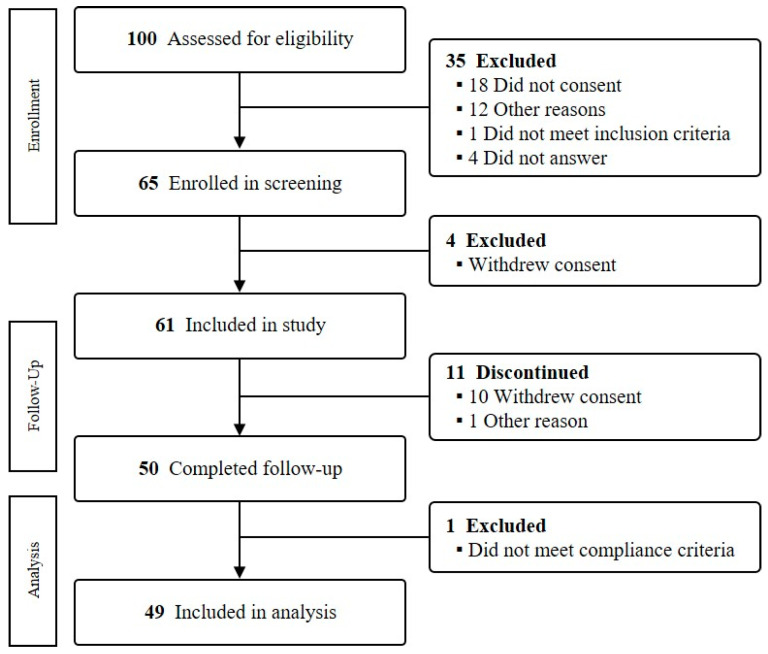
Flowchart of the study.

Study participants were recruited as community volunteers through the E.Jo Connection, a recruitment agency for clinical trials, and the community advertisement near the Ewha Clinical Trial Center of Mok-dong Hospital. Women aged over 50 and under 65 were recruited. To evaluate the intervention outcomes in general middle-aged women, disease history or current symptoms were not included in the exclusion criteria. At the screening step, the individual intake amount of four major nutrients was estimated via the dietary survey using 24 h recall on 3 days (2 weekdays and 1 weekend day). According to the nutritional intake evaluation, all subjects showed a low intake level in at least one of the target nutrients below Korean dietary recommendations (75 mg of isoflavone, 2.0 g of omega-3 fatty acid, 18.8 g of fiber, and 600 mg of calcium). Subjects who were hypersensitive to certain foods or ingredients or had difficulty using smartphones were excluded. Among the 65 enrolled subjects, 61 participated in the intervention (Figure 1). A total of 50 completed the intervention (11 subjects withdrew their consent or stopped for personal reasons). According to dietary compliance, a total of 49 subjects who had over 70% of compliance were included in further analyses. The compliance calculation method was as follows: (number of meals consumed/total number of meals provided) × 100.

### 2.2. Dietary Intervention and Assessment

Analysis of the current dietary intake status of Korean middle-aged women was conducted using the dietary intake data from the 2016 and 2017 Korean National Health and Nutrition Examination Survey (women aged between 50 to 80 years old, *n* = 3440; data not shown,; available on request) to screen the main target nutrients for intervention. Low intake nutrients on average, compared to the recommended nutrient intake or adequate intake for the Korean population [26], were identified and narrowed down to four nutrients (calcium, omega-3, fiber, and isoflavone), which are frequently applied for dietary intervention in middle-aged women [27].

Registered dietitians designed the meal plans for two weeks, which were run four times during an 8-week intervention. The meals were served as 2 meals/day (lunch, dinner) and 1 snack under planning to provide approximately 80% of the estimated energy requirement per day and the according macronutrients, and sufficient four target nutrients according to Korean Dietary Reference Intake [26]. The meals were provided in the form of meal kits with recipe card. Participants were instructed to prepare and consume meals at regular times according to each individual’s eating schedule with a simple breakfast meal being recommended.

The usual dietary behavior was surveyed using two different approaches: the Recommended Food Score (RFS); and Meats, Eggs, Dairy, Fried foods, fat In baked foods, Convenience foods, fats added at Table, and Snacks (MEDFICTS). The RFS is a food-based score that assesses diet quality, as suggested by Kant et al. [28], and the RFS modified for the Korean diet was used [29]. MEDFICTS was investigated for dietary fat and cholesterol consumption. At 0 week, before starting the intervention, participants were educated on the dietary and lifestyle guidelines. During the intervention phase, nutritional intake was recorded using the smartphone application E-diary II (Biofood Co., Ltd., Seoul, Republic of Korea) and monitored by researchers every week. Intervention was completed in the range of 82 to 112 meals per person. The average amount of nutrients per day was as follows: 1531.7 kcal of energy, 182.1 mg of isoflavone, 11.9 g of omega-3, 25.3 g of fiber, and 833.4 mg of calcium.

### 2.3. Measurements

Demographic characteristics, including birth date, residence, age, pregnancy, and menopausal status, were obtained before the intervention. Menopause was defined as at least 12 months of amenorrhea, and a shorter period was designated as perimenopause. Participants visited the hospital after 12 h of fasting in week 0 and week 8 and were examined using the following tests: (1) anthropometric parameters (e.g., height, body weight, body mass index (BMI), waist circumference, hip circumference, systolic blood pressure, and diastolic blood pressure); (2) body composition (e.g., body water, body fat, skeletal muscle, and abdominal fatness); and (3) biochemical parameters, including TC, triglyceride (TG), high-density lipoprotein-cholesterol (HDL-C), LDL-C, lipoprotein(a) (Lp(a)), apoprotein A1 (ApoA1), apoprotein B (ApoB), glucose, insulin, high-sensitivity C-reactive protein (hs-CRP), E2, FSH, C-terminal telopeptide of type I collagen (CTx), and osteocalcin. Body composition was measured using the InBody 720 (Biospace Co., Seoul, Republic of Korea), and the skeletal muscle index (SMI) was calculated using the following formula: [appendicular lean mass (kg)/body weight (kg)] × 100. Most blood parameters were analyzed at the Ewha Womans University Mok-dong Hospital, ApoB was analyzed by GCCL (Yongin, Republic of Korea), CTx was analyzed by EONE Laboratories (Incheon, Republic of Korea), and osteocalcin was analyzed by Seegene, Inc. (Seoul, Republic of Korea). Briefly, the concentrations of glucose and TC were estimated using the COD-POD method, and TG was calculated using the GPO-PAP method. The selective inhibition enzymatic method was used for HDL-C and LDL-C, and turbidimetry was conducted for ApoA1, ApoB, and LP(a) detection. The hormones, including insulin, E2, and osteocalcin, were estimated using electrochemiluminescence microparticle immunoassay, and FSH and CTx were calculated using chemiluminescence microparticle immunoassay and chemiluminescence immunoassay, respectively.

### 2.4. Menopausal Index

The Kupperman Index (KI), a self-report method for clinical evaluation of menopausal symptoms in menopausal women [30], was measured to examine the menopausal symptoms between pre- and post-intervention. The modified KI consisting of 12 symptoms (hot flashes/sweating, paresthesia, insomnia, nervousness, depression, vertigo, fatigue, arthralgia/myalgia, headache, palpitation, itching, and vaginal dryness) was used in the study. The severity of each symptom can be selected on a scale between 0 and 3 points, and the total score range was set from 0 to 54 points with 4 times the weight of hot flashes/sweating (0 to 12 points) and multiplied by 2 times the weight of paresthesia, insomnia, and nervousness (0 to 6 points). To measure the improvement in sleep disorders, the Pittsburgh Sleep Quality Index (PSQI) and Insomnia Severity Index (ISI) were examined [31].

### 2.5. Statistical Analysis

For continuous variables including nutritional intake, menopausal symptoms, body measurement, body composition, and biochemical indicators, the paired *t*-test was used to compare the parameters in the pre- and post-intervention phases. Additionally, Cohen’s d was calculated for measurement of effect size. Categorical values were analyzed using the chi-square test. All statistical analyses were calculated using SAS version 9.4 (SAS Institute, Inc., Cary, NC, USA) and R software version 4.1.3 (Rstudio Inc., Boston, MA, USA). A *p* < 0.05 was initially considered to be statistically significant and Bonferroni correction was applied to correct multiple testing (Bonferroni correction *p* < 0.002).

## 3. Results

### 3.1. General Characteristics of Participants

The basic characteristics of the participants are shown in Table 1. The average age of the participants was 58.20 ± 4.24 years, and the average height was 156.34 ± 5.37 cm. Forty-three participants (87.8%) were post-menopausal and 5 participants (10.2%) were perimenopausal. Four of them were under hormonal therapy. The average sleep duration was 6.31 ± 1.16 h, and the ISI score, an index of sleep quality evaluation, was 8.96 ± 5.73 points, indicating subthreshold insomnia, which ranges between 8 and 14 points [31].

There were no smokers, and 18 participants (36.7%) were current drinkers. The MEDFICTS score was 44.82 ± 24.47 points, which included the Step 1 diet. The average RFS of the participants was 25.80 ± 5.93 points. Regarding drug use, 11 (22.4%) were using hypertension drugs, 15 (30.5%) were using hyperlipidemia drugs, 1 (2%) was using antidepressant drugs, 8 (16.3%) were using sleep-inducing drugs, 1 (2%) was using dietary supplements, and 19 (38.8%) were using drugs for other reasons (e.g., menopausal symptoms, diabetes, and osteoporosis).

### 3.2. Nutrient Intake Analysis

To compare pre- and post-intervention dietary nutrient intake, the nutrient intake was analyzed (Table 2). The intervention significantly increased the average daily intake of dietary fiber (4.92 ± 6.71 g), resulting in 19.96 ± 4.26 g of fiber intake post-intervention (*p* < 0.0001). Calcium increased by 354.50 ± 159.20 mg, from 332.61 ± 152.60 mg pre-intervention to 687.11 ± 170.30 mg post-intervention (*p* < 0.0001), while the intake of sodium, which is involved in renal calcium reabsorption, was not changed by the intervention (*p* = 0.799). Isoflavone intake increased from 13.14 ± 9.55 mg pre-intervention to 52.81 ± 18.45 mg post-intervention (*p* < 0.0001). Additionally, among the omega-3 fatty acids, the intake of eicosapentaenoic acid was significantly elevated (46.85 ± 73.13 mg, *p* < 0.0001). These results indicate that the dietary intervention effectively increased the intake of the target nutrients; in particular, the average dietary fiber intake and calcium intake were higher than the recommended amounts for middle-aged women (18.8 g for dietary fiber and 600.0 mg for calcium).

Interestingly, the intervention increased the daily energy intake (303.79 ± 416.90 kcal, *p* < 0.0001), accompanied by an increased intake of macronutrients. Specifically, carbohydrate intake was increased by 23.16 ± 57.35 g, from 192.56 ± 56.09 g pre-intervention to 215.71 ± 38.79 g post-intervention (*p* = 0.007). Protein intake was increased by 24.06 ± 19.46 g, and fat intake was increased by 12.43 ± 19.36 g (*p* < 0.0001 for both). The intervention changed the percentages of total energy intake of macronutrients, with a decrease in carbohydrates and an increase in protein and fat (carbohydrate–protein–fat ratio of 60:16:24 pre-intervention and 55:19:26 post-intervention). Average intakes of cholesterol and saturated fatty acid showed no significant changes (*p* = 0.167 and *p* = 0.734, respectively).

### 3.3. Changes in Anthropometric and Body Composition Parameters Induced by Intervention

Despite an increased dietary energy intake, there were favorable changes in anthropometric measurements (Table 3). After 8 weeks, body weight was significantly decreased by −0.52 ± 1.17 kg (*p* = 0.003); however, its significance was diminished after the Bonferroni correction with small effect size (*d* = 0.447). BMI and waist circumference were decreased by −0.16 ± 0.49 kg/m^2^ (*p* = 0.025) and −1.27 ± 5.00 kg (*p* = 0.083). There were no differences between the pre- and post-intervention systolic and diastolic blood pressures (*p* = 0.816 and *p* = 0.501, respectively). Regarding body composition, the body fat was reduced by −0.30 ± 1.04 kg, from 20.38 ± 4.40 kg pre-intervention to 20.08 ± 4.65 kg post-intervention (*p* = 0.049), while skeletal muscle mass and SMI were not changed.

### 3.4. Alterations in Biochemical Parameters

CTx and osteocalcin, two indicators of osteoporosis risk in postmenopausal women, were decreased by −0.03 ± 0.09 ng/mL (*p* = 0.037) and −2.08 ± 3.09 ng/mL (*p* < 0.0001); particularly, the change in osteocalcin was significant even after the Bonferroni correction with medium effect size (*d* = 0.306), implying a reduced bone loss post intervention (Table 4). Regarding changes in hormone profiles, FSH showed a marginal reduction (*p* = 0.059), whereas E2 was not affected (*p* = 0.312). Positive alterations in blood lipid profile were detected, with decreased levels of TC (−10.08 ± 30.51 mg/dL, *p* = 0.025) and LDL-C (−10.06 ± 26.81 mg/dL, *p* = 0.012). Moreover, the ratios of LDL-C/HDL-C and TC/HDL-C, which are known as CVD predictive indicators, were reduced post intervention (*p* = 0.009 and *p* = 0.025, respectively). ApoA1 and ApoB, which are associated with HDL and LDL, respectively [32], were significantly decreased with medium effect (*p* < 0.0001, *d* = 0.673 and *p* = 0.001, *d* = 0.502, respectively), whereas there was no significant change in the ratio of ApoB/ApoA1 (*p* = 0.955). Among the blood glycemic parameters, the level of insulin was significantly reduced (*p* = 0.040), and the level of glucose was marginally decreased (*p* = 0.065) post intervention.

### 3.5. Improvement in KI and PSQI

The severity of menopausal symptoms in the pre-intervention phase belonged to the mild level, with 14.27 ± 8.18 points of KI score. The KI score was significantly decreased by −3.53 ± 5.07 points and the change was significant even after multiple comparison correction with medium effect size (*p* < 0.0001 and *d* = 0.697); in addition, the PSQI score was reduced by −0.55 ± 1.78 points (*p* = 0.018), indicating alleviation of menopausal symptoms and improvement of sleep quality post intervention (Table 5).

## 4. Discussion

In the past few years, research on promoting the health status of postmenopausal women has focused on supplementation with functional compounds. In this pilot study, the potential benefits of a meal-based intervention on the health promotion of middle-aged women were investigated by developing a meal plan enriched with target nutrients for menopausal women. Decreases in BMI and body fat, and improvement in menopausal symptoms and sleep quality were detected after dietary intervention. Moreover, parameters for bone metabolism, blood lipid profiles, and glycemic regulation were improved post intervention. Reduction in KI, osteocalcin, and Apo proteins levels was significant, even after adjustment for multiple comparison.

Recently, the focus of nutritional epidemiological investigations has moved beyond individual nutrient approaches to the broader perspective of whole diets [33] due to the complex interactions between nutrients and cumulative effects of food intake with low safety risk [34]. Various studies show that dietary interventions modulating nutrients have beneficial impacts on several metabolic diseases, including obesity, type 2 diabetes, and hypertension, which can cause various complications [35,36,37]. Given that diet directly affects systemic metabolism, including blood glucose and lipid concentrations [38], diet should be considered a prominent strategy. Moreover, as a strong driver of microbiome, diets can modify the gut microbiota composition and lead to changes in the end-products of foods, resulting in favorable health outcomes [39,40]. However, due to the cost, time, and physical limitations of providing an entire meal, most previous studies focusing on menopausal women focused on plant-based supplements or nutrient-enhanced drugs. Eating a meal containing abundant nutrients induces potential synergy by affecting the absorption mechanism and rate of nutrients, whereas approaches that focus on individual nutrients might not be able to consider its effectiveness and relationship with other nutrients [25]. Moreover, dietary intervention could prevent the adverse effects produced by functional components. For instance, with fiber, which is one of the major nutrients of the present study, supplementations with various types of fiber products improved constipation but caused gastrointestinal symptoms including abdominal pain [41]. Furthermore, some studies have showed little-to-no benefits or harmful impacts at high doses of functional components [42,43,44,45]. A cross-over clinical research using two fiber products has revealed that there was an individual range of response to arabinoxylan and a high-dose intake of inulin led to inflammation and liver damage [42]. Most national recommendations, including those in Korea, lack information regarding the consumption of each functional component. These results have shown that types of functional component and dose regime should be considered when functional components are supplemented. Furthermore, recognizing regular eating habits by recording nutritional intake during interventions and improving knowledge of nutrition and health can induce long-term changes toward a desirable diet, leading to health promotion [46,47].

In this study, favorable changes in body weight, BMI, and waist circumference, the improvement of blood lipid, glucose profiles, and bone resorption markers were detected after the intervention. Due to the function of sex hormones in regulating metabolism and sex-specific remodeling of fat cells, the decrease in estrogen in menopausal women induces abdominal central fat gain and body weight gain [48]. In addition, studies have shown that increased FSH induced by ovarian aging positively correlates with body weight [49]. Changes in hormones and body mass in menopause were strongly associated with lipid profile alterations. In post-menopausal women, ApoB, which is involved in LDL metabolism, was increased [50], and the serum TC and LDL levels were positively correlated with FSH [51]. Moreover, high concentrations of free fatty acids, LDL, and ApoB were associated with increased abdominal fat, resulting in an increased risk of CVD [52,53]. These symptoms were alleviated by nutrient supplementation. Omega-3 supplementation of 900 mg/day in 87 postmenopausal women significantly reduced BMI, waist circumference, TG levels, and interleukin-6 concentrations and improved insulin resistance [12]. In addition, calcium intake was negatively related to body weight, body fat, blood glucose, and TG level in middle-aged women [54]. In a two-year randomized controlled study of 500 healthy postmenopausal women, calcium-rich milk effectively attenuated bone resorption and reduced fasting blood glucose, hemoglobin A1c, TC, LDL, and ApoB levels [55]. Providing various plant-derived isoflavone products to menopausal women for 6 months significantly decreased their KI scores and the TC, LDL, and TG levels [56]. One study, which investigated the impacts of 12-week supplementation of isoflavone and γ-linolenic acid compound in Korean menopausal women, reported decreased KI score and oxidative LDL concentration but no improvement in the TC, LDL, and ApoB concentrations [57]. These results suggest that the potential benefits of our dietary intervention on body weight loss and improvement in blood lipid profile in menopausal women might also be a valid program to reduce the risk of CVD.

Menopause is a discontinuation of ovarian activity that causes various symptoms due to changes in sex hormones [1]. The modified KI is a self-rating score that covers 12 aspects of menopausal symptoms. A decrease in the KI score indicates multiple hormonal alterations and physical and metabolic symptoms in menopausal women [58]. In our study, the reinforced dietary intake of target nutrients, such as isoflavone and omega-3, decreased the KI and PSQI, although changes in FSH were not obvious upon intervention. Hot flashes and sweating, two representative symptoms of menopause, are associated with a significantly high FSH level and low E2 level in menopause [52,59,60]. The increase in the level of FSH contributes to vascular calcification and arteriosclerosis by producing foam cells, accumulating lipids in the endothelium of vascular cells, stimulating T cells, and releasing inflammatory cytokines [49,52]. Excessive production of cytokines can induce vascular dysfunction, depression, and fatigue stress in menopausal women [61]. In addition, hormonal changes, such as the lack of estrogen, are considered one of the complex factors of insomnia in menopausal women [62]. Omega-3 fatty acids improved hot flashes and depression in menopausal women [63], and isoflavone supplementation attenuated insomnia symptoms in post-menopausal women [64]. In addition, in a randomized placebo-controlled study, isoflavone or soybean extract supplementation reduced KI scores and the number and severity of facial flushes in menopausal women [23,57,65,66]. However, in some studies, isoflavone supplementation for 12 weeks did not improve the KI score or QoL in menopausal women after 12 weeks [44,45]. It is noteworthy that the changes in the KI score in our study were only from 14 down to 11; however, it was statistically significant, and could therefore not be clinically implied. However, the limited changes might have been due to characteristics of the participants, who were low-symptomatic in the pre-intervention phase with a mild range of KI scores. The results suggested that the meal-based intervention might be an effective strategy to improve overall health parameters as regards middle-aged women and further investigation is required for addressing its efficacy on menopausal symptoms.

In the present pilot study, the results suggested the potential benefits of meal-based intervention. However, there are several points to consider for implementation of the intervention in a larger study. Firstly, the present pilot study adopted a comparison between the pre- and post-intervention phases without a control group or crossover design. The design carried limitations on feasibility, although the effect size was measured to estimate the feasibility and the Bonferroni correction was applied for interpretation of the data. Although subjects were instructed not to change their regular eating schedules and physical activity during the study period, we could not directly control such lifestyle factors. A further larger-scale study including a control group and double-blinded design is needed to confirm the potential effectiveness of our model. Secondly, an improved method of participant recruitment should be sought in order to obtain clearer results. This study was based on the general population regardless of their status of metabolic and menopausal parameters. To test the efficacy of this model, it might be necessary to recruit subjects with certain symptoms and metabolic statuses. Thirdly, meal-based intervention providing meal kits is characterized by cooking the food, which means that the lifestyle of participants should be considered. Although all subjects received instructions on meal intake, the timing of meal intake and meal skipping may be affected by occupational status or the individual’s regular eating schedule. Fourthly, our intervention design showed a relatively high retention rate of participants. Among the 61 participants enrolled, only 1 was excluded due to a dietary compliance issue, suggesting high acceptability of intervention. The others were excluded for personal reasons or difficulty using devices (application and blood glucose meter). These results show that the convenience and good quality of the diet provided in the intervention study led to high compliance, which means that the intervention is feasible for a large population, and consideration of the use of instruments may be necessary for a higher retention. Finally, qualitative data including questionnaires and interviews should be collected to complement this pilot study and additional long-term follow-up data might be useful to demonstrate the sustainability of the intervention.

## 5. Conclusions

After the meal-based precision intervention, menopausal symptoms, bone metabolism, and blood glucose and lipid profiles were improved, resulting in favorable health outcomes in relatively healthy menopausal women. These results suggested that meal-based interventions specifically designed for middle-aged women might be an effective strategy for health promotion.

## Figures and Tables

**Table 1:** General characteristics of participants.

Characteristics	Participants (*n* = 49)
Age, years	58.20 ± 4.24
Height, cm	156.34 ± 5.37
Physical activity, kcal/day	265.36 ± 205.94
Menopause	
Post-menopause	43 (87.80)
Perimenopause	5 (10.20)
Pre-menopause	1 (2.00)
Sleeping history	
Sleeping time, h	6.31 ± 1.16
ISI *	8.96 ± 5.73
No insomnia	22 (44.90)
Subthreshold insomnia	15 (30.61)
Clinical insomnia	12 (24.49)
Alcohol drinking habit	
Current	18 (36.70)
Dietary history	
MEDFICTS **	44.82 ± 24.47
Step 2 diet	24 (48.98)
Step 1 diet	19 (38.78)
RFS ***	25.80 ± 5.93
Medication history	
Antihypertensive agents	11 (22.40)
Lipid-lowering medication	15 (30.60)
Antidepressants, sedatives, anxiety pills	1 (2.00)
Sleeping pills	8 (16.30)
Dietary supplements	1 (2.00)
Other drugs	19 (38.80)

Data are represented as mean ± standard deviations or number (%). * Insomnia Severity Index (ISI): (0–7: no insomnia, 8–14: subthreshold insomnia, 15–21: clinical insomnia, 22–28: severe clinical insomnia); ** Meats, Eggs, Dairy, Fried foods, fat In baked goods, Convenience foods, fats added at Table, and Snacks (MEDFICTS): (<40 points: Step 2 diet [7% of energy from saturated fat and <200 mg cholesterol], 40–69 points: Step 1 diet [8–10% of energy from saturated fat and <300 mg cholesterol], >70 points: high-fat diet); *** Recommended Food Score (RFS): The scores range from 0 to 47, and the higher score indicates the better quality of the diet.

**Table 2:** Daily nutrient and energy intakes at baseline and after 8 weeks of intervention.

Variables	Pre	Post	Change	*p*-Value
Targeted nutrients				
Fiber (g)	15.05 ± 5.27	19.96 ± 4.26	4.92 ± 6.71	<0.0001
Calcium (mg)	332.61 ± 152.60	687.11 ± 170.30	354.50 ± 159.20	<0.0001
Isoflavones (mg)	13.14 ± 9.55	52.81 ± 18.45	39.67 ± 22.62	<0.0001
Omega-3 fatty acids (g)	1.09 ± 0.62	1.17 ± 0.31	0.08 ± 0.60	0.367
α-Linolenic acid 18:3 (mg)	819.73 ± 533.52	836.90 ± 279.66	17.17 ± 589.40	0.841
Stearidonic acid 18:4 (mg)	14.11 ± 18.39	11.38 ± 8.63	−2.73 ± 17.85	0.295
Eicosapentaenoic acid 20:5 (mg)	86.49 ± 69.48	133.34 ± 49.36	46.85 ± 73.13	<0.0001
Docosahexaenoic acid 22:6 (mg)	139.37 ± 122.10	161.92 ± 76.44	22.55 ± 116.60	0.187
Macronutrients				
Energy (kcal)	1273.48 ± 385.55	1577.27 ± 259.82	303.79 ± 416.90	<0.0001
Carbohydrate (g)	192.56 ± 56.09	215.71 ± 38.79	23.16 ± 57.35	0.007
Protein (g)	50.14 ± 16.59	74.19 ± 13.42	24.06 ± 19.46	<0.0001
Fat (g)	34.29 ± 15.72	46.71 ± 11.22	12.43 ± 19.36	<0.0001
Other nutrients				
Sodium (mg)	2552.34 ± 1252.88	2595.81 ± 580.40	43.47 ± 1177.80	0.799
Cholesterol (mg)	268.56 ± 119.32	294.33 ± 80.40	25.77 ± 127.10	0.167
Saturated fatty acid (g)	11.04 ± 5.78	10.67 ± 7.09	−0.38 ± 7.60	0.734

Data are represented as mean ± standard deviations. (*n* = 48; one of the participants was excluded because of data loss).

**Table 3:** Changes in anthropometric measurement and body composition according to intervention.

Variables	Pre	Post	Change	*p*-Value	Effect Size
Anthropometric parameters					
Weight (kg)	57.98 ± 6.72	57.46 ± 6.67	−0.52 ± 1.17	0.003	0.447
BMI (kg/m^2^)	23.69 ± 2.21	23.52 ± 2.22	−0.16 ± 0.49	0.025	0.330
Waist circumference (cm)	79.55 ± 6.24	78.29 ± 6.48	−1.27 ± 5.00	0.083	0.253
Hip circumference (cm)	93.81 ± 3.82	94.17 ± 4.83	0.36 ± 4.00	0.528	0.091
Systolic blood pressure (mmHg)	124.45 ± 15.21	124.86 ± 13.41	0.41 ± 12.20	0.816	0.033
Diastolic blood pressure (mmHg)	72.49 ± 11.70	71.55 ± 8.94	−0.94 ± 9.69	0.501	0.097
Body composition					
Total body water (L)	27.63 ± 3.11	27.46 ± 3.07	−0.17 ± 0.73	0.119	0.227
Body fat (kg)	20.38 ± 4.40	20.08 ± 4.65	−0.30 ± 1.04	0.049	0.288
Total body fat mass (%)	34.93 ± 4.81	34.70 ± 5.31	−0.23 ± 1.50	0.282	0.155
Abdominal fatness (%)	0.85 ± 0.03	0.85 ± 0.04	0.00 ± 0.02	0.646	0.430
Skeletal muscle mass (kg)	20.22 ± 2.56	20.10 ± 2.52	−0.11 ± 0.56	0.168	0.200
SMI (%)	26.39 ± 2.16	26.33 ± 2.36	−0.06 ± 0.60	0.502	0.097

Data are represented as mean ± standard deviations. Asterisk (*) indicates if the *p*-value was significant even after Bonferroni correction for multiple comparisons (corrected *p*-value: 0.05/31 = 0.002). BMI, Body mass index; SMI, Skeletal muscle index.

**Table 4:** Biochemical parameters before and after the 8-week intervention.

Variables	Pre	Post	Change	*p*-Value	Effect Size
Bone turnover markers					
CTx (ng/mL)	0.40 ± 0.16	0.37 ± 0.14	−0.03 ± 0.09	0.037	0.306
Osteocalcin (ng/mL)	16.33 ± 4.84	14.25 ± 4.46	−2.08 ± 3.09	<0.0001 *	0.673
Hormones					
E2 (pg/mL)	35.33 ± 154.83	12.90 ± 22.99	−22.43 ± 153.80	0.312	0.146
FSH (mIU/mL)	62.52 ± 31.30	59.75 ± 30.46	−2.77 ± 10.02	0.059	0.277
Lipid profile					
TG (mg/dL)	120.65 ± 67.47	112.06 ± 106.28	−8.59 ± 80.68	0.460	0.106
TC (mg/dL)	209.78 ± 39.98	199.69 ± 43.38	−10.08 ± 30.51	0.025	0.330
HDL-C (mg/dL)	58.88 ± 13.62	59.92 ± 13.27	1.04 ± 8.78	0.411	0.119
LDL-C (mg/dL)	134.69 ± 37.13	124.63 ± 37.34	−10.06 ± 26.81	0.012	0.375
LDL-C/HDL-C	2.41 ± 0.84	2.16 ± 0.77	−0.25 ± 0.63	0.009	0.387
TC/HDL-C	3.74 ± 1.09	3.46 ± 1.01	−0.28 ± 0.84	0.025	0.330
hs-CRP (mg/dL)	0.08 ± 0.08	0.12 ± 0.17	0.04 ± 0.16	0.113	0.230
Lp(a) (mg/dL)	21.70 ± 20.58	21.99 ± 19.78	0.28 ± 5.35	0.706	0.054
ApoA1 (mg/dL)	161.50 ± 22.03	149.19 ± 23.98	−12.31 ± 18.29	<0.0001 *	0.673
ApoB (mg/dL)	104.65 ± 24.39	96.20 ± 22.09	−8.45 ± 16.82	0.001 *	0.502
ApoB/ApoA1	1.65 ± 0.54	1.65 ± 0.54	0.001 ± 0.014	0.955	0.001
Glycemic parameters					
Glucose (mg/dL)	95.76 ± 15.62	91.31 ± 7.95	−4.45 ± 16.47	0.065	0.270
Insulin (μU/mL)	8.56 ± 5.16	7.06 ± 3.47	−1.50 ± 4.98	0.040	0.301

Data are represented as mean ± standard deviations. Asterisk (*) indicates if the *p*-value was significant even after Bonferroni correction for multiple comparisons (corrected *p*-value: 0.05/31 = 0.002). CTx, C-terminal telopeptide of type I collagen; E2, Estradiol; FSH, Follicle-stimulating hormone; TG, Triglyceride; TC, Total cholesterol; HDL-C, High-density lipoprotein cholesterol; LDL-C, Low-density lipoprotein cholesterol; LP(a), Lipoprotein(a); ApoA1, Apoprotein A1; ApoB, Apoprotein B.

**Table 5:** Assessment of postmenopausal symptoms at baseline and intervention.

Variables		Pre	Post	Change	*p*-Value	Effect Size
KI		14.27 ± 8.18	10.73 ± 7.00	−3.53 ± 5.07	<0.0001 *	0.697
	No complaint	9 (18.37)	15 (30.61)	3.7315 ^†^	0.292	
	Mild	25 (51.02)	22 (44.90)			
	Moderate	13 (26.53)	12 (24.49)			
	Severe	2 (4.08)	0 (0)			
PSQI		5.61 ± 2.08	5.06 ± 2.21	−0.55 ± 1.78	0.018	0.310

Data are represented as mean ± standard deviations or number (%). Dagger (^†^) represents the chi-square value. Asterisk (*) indicates if the *p*-value was significant even after Bonferroni correction for multiple comparisons (corrected *p*-value: 0.05/31 = 0.002). Kupperman index (KI): ‘no complaint’ (score 0–6), ‘mild’ (score 7–15), ‘moderate’ (score 16–30), or ‘severe’ (score > 30); Pittsburgh Sleep Quality Index (PSQI): score range is 0–21, and a score of ≥5 points denotes poor sleep quality.

## Data Availability

Data sharing is not applicable to this article.
